# *Drosophila *larvae lacking the *bcl-2 *gene, *buffy*, are sensitive to nutrient stress, maintain increased basal target of rapamycin (Tor) signaling and exhibit characteristics of altered basal energy metabolism

**DOI:** 10.1186/1741-7007-10-63

**Published:** 2012-07-24

**Authors:** Jessica P Monserrate, Michelle Y-Y Chen, Carrie Baker Brachmann

**Affiliations:** 1 Developmental and Cell Biology, University of California, Irvine, CA 92697, USA

**Keywords:** autophagy, Bcl-2, metabolism, non-apoptotic, nutrient restriction, S6K, starvation, Tor

## Abstract

**Background:**

B cell lymphoma 2 (Bcl-2) proteins are the central regulators of apoptosis. The two *bcl-2 *genes in *Drosophila *modulate the response to stress-induced cell death, but not developmental cell death. Because null mutants are viable, *Drosophila *provides an optimum model system to investigate alternate functions of Bcl-2 proteins. In this report, we explore the role of one *bcl-2 *gene in nutrient stress responses.

**Results:**

We report that starvation of *Drosophila *larvae lacking the *bcl-2 *gene, *buffy*, decreases survival rate by more than twofold relative to wild-type larvae. The *buffy *null mutant reacted to starvation with the expected responses such as inhibition of target of rapamycin (Tor) signaling, autophagy initiation and mobilization of stored lipids. However, the autophagic response to starvation initiated faster in larvae lacking *buffy *and was inhibited by ectopic *buffy*. We demonstrate that unusually high basal Tor signaling, indicated by more phosphorylated S6K, was detected in the *buffy *mutant and that removal of a genomic copy of S6K, but not inactivation of Tor by rapamycin, reverted the precocious autophagy phenotype. Instead, Tor inactivation also required loss of a positive nutrient signal to trigger autophagy and loss of both was sufficient to activate autophagy in the *buffy *mutant even in the presence of enforced phosphoinositide 3-kinase (PI3K) signaling. Prior to starvation, the fed *buffy *mutant stored less lipid and glycogen, had high lactate levels and maintained a reduced pool of cellular ATP. These observations, together with the inability of *buffy *mutant larvae to adapt to nutrient restriction, indicate altered energy metabolism in the absence of *buffy*.

**Conclusions:**

All animals in their natural habitats are faced with periods of reduced nutrient availability. This study demonstrates that *buffy *is required for adaptation to both starvation and nutrient restriction. Thus, Buffy is a Bcl-2 protein that plays an important non-apoptotic role to promote survival of the whole organism in a stressful situation.

## Background

Mammalian B cell lymphoma 2 (Bcl-2) proteins are the central regulators of apoptosis (reviewed in [1]). Cells die when the activity of proapoptotic Bcl-2 proteins exceeds that of antiapoptotic Bcl-2 proteins. The detrimental effects of deleting *bcl-2 *family members has been historically attributed to loss of apoptotic activity, but there is growing evidence that at least some of the Bcl-2 family members play an active, non-apoptotic role in maintaining cellular health [2]. In fact, many apoptotic mediators have a dual role. For example, cytochrome *c *is essential for respiration, a pro-survival function, as well as activation of apoptotic protease activating factor 1 (Apaf-1) and caspases to promote death. Apoptosis inducing factor (AIF) and mitochondrial fission factors such as dynamin-related protein 1 (Drp1)/dynamin 1 (Dnm1) also play dual roles in cells [3-6]. These findings are leading researchers to look more carefully at the biology of apoptotic regulators in normal cells. Bcl-2 family proteins have biological functions in the endoplasmic reticulum (ER) to mitochondrial calcium signaling, mitochondrial dynamics, the unfolded protein response, autophagy, cell cycle control and mitochondrial energy metabolism (reviewed in [7]). Although these pathways may seem quite varied at first glance, they all respond to stress conditions, such as DNA damage, metabolic dysregulation, unfolded proteins, hypoxia and growth factor withdrawal. Active participation of Bcl-2 proteins in these stress-responsive pathways allows these apoptotic regulators to initiate cell death following a loss in cellular homeostasis. Thus, the 'day jobs' of the Bcl-2 proteins safeguard the health of the multicellular organism, whilst their more glamorous roles are in apoptosis.

The canonical apoptotic pathway, regulated by the Bcl-2 proteins, leads to activation of caspases upon release of proapoptotic factors from the mitochondria. This mitochondrial pathway for apoptosis is not strictly conserved in flies and worms, nevertheless Bcl-2 proteins are found in these metazoans, function in cell death *in vivo *and can substitute for mammalian Bcl-2 proteins [8-10]. In the case of *Drosophila*, unlike mammals or worms, the Bcl-2 proteins are not involved in regulating developmental programmed cell death (PCD), but modulate the apoptotic response to stress [11-13]. Whether release of mitochondrial factors is essential for fruit fly apoptosis is still unclear, but many of the known *Drosophila *apoptotic molecules, including the Bcl-2 proteins, are found at the mitochondria and recent data indicates a role for mitochondrial fission in *Drosophila *cell death [14-19].

The present study grew out of an observation that *Drosophila *lacking *buffy*, one of the two *bcl-2 *genes in fruit flies, were unable to withstand nutrient stress. Most animals face periods of reduced nutrient availability in their natural habitats and metabolically adapt to allow starvation resistance [20]. Because *Drosophila *larvae must feed continuously to maintain growth and store nutrients for metamorphosis, even a few hours of amino-acid starvation induces a starvation response [21,22]. When larvae are subjected to nutrient withdrawal, growth slows to reduce energy requirements and the catabolic process of autophagy is activated [22].

Glycogen and lipids are energy sources that are utilized when animals face nutrient stress. In mammals, these energy sources are stored in liver and adipose tissue and their metabolism is regulated by the cyclic AMP (cAMP) responsive element binding (CREB) protein. *Drosophila *have a single organ, the fat body, that is akin to the mammalian liver and adipose tissue, and glycogen and lipid storage and mobilization are similarly regulated through the *Drosophila *CREB homolog [23]. *Drosophila *populations that are selected for starvation resistance have a genetically determined increase in both lipid and carbohydrate storage [24]. Metabolism alterations that favor survival over growth are also observed in starved animals. The molecular mechanisms of such a shift in metabolism are not well understood, but result in reduced energy demands [25].

The conserved Ser/Thr kinase family of target of rapamycin (Tor) proteins mediates growth in response to nutrients, energy and growth factor signals [26-28]. Loss of these signals, or cellular stress, rapidly inhibits Tor signaling with the result that growth slows and autophagy begins. Intracellular amino acids regulate Tor activity through the Ragulator complex that promotes translocation of Tor to peripheral lysosomal membranes where Rheb can activate Tor kinase activity. Nutrient starvation blocks Tor activation by disabling shuttling of Tor to this Rheb compartment, in contrast to rapamycin that blocks Tor activity through direct binding and inhibition of Tor kinase activity [29-31]. Tor activity is also negatively regulated by cellular energy levels through the energy-sensing kinase AMP-activated protein kinase (AMPK).

We report that animals lacking *buffy *have characteristics of altered basal energy metabolism and increased Tor signaling. *buffy *mutant larvae are sensitive to nutrient restriction and prolonged starvation, but are not deficient in starvation responses. However, starvation-induced autophagy was activated faster in larvae lacking *buffy*. Removal of one copy of S6K reverted this phenotype, but ectopic activation of phosphoinositide 3-kinase (PI3K) signaling did not, suggesting that both the altered basal metabolism and increased phosphorylated S6K are required for this phenotype.

## Results

We observed that *buffy *null mutants were sensitive to crowded vials or poor food quality. In such challenging growing conditions, *buffy *null larvae grew slower and were often smaller than their wild-type counterparts, and *buffy *null pupae had reduced eclosure rates. In contrast, when grown in non-crowded, nutrient rich conditions, larvae lacking *buffy *were indistinguishable from wild-type larvae and pupae eclosed at normal rates. These observations suggested that Buffy regulates the response to reduced nutrients.

### Larvae lacking *buffy *are sensitive to acute nutrient deprivation

**Figure 1:**
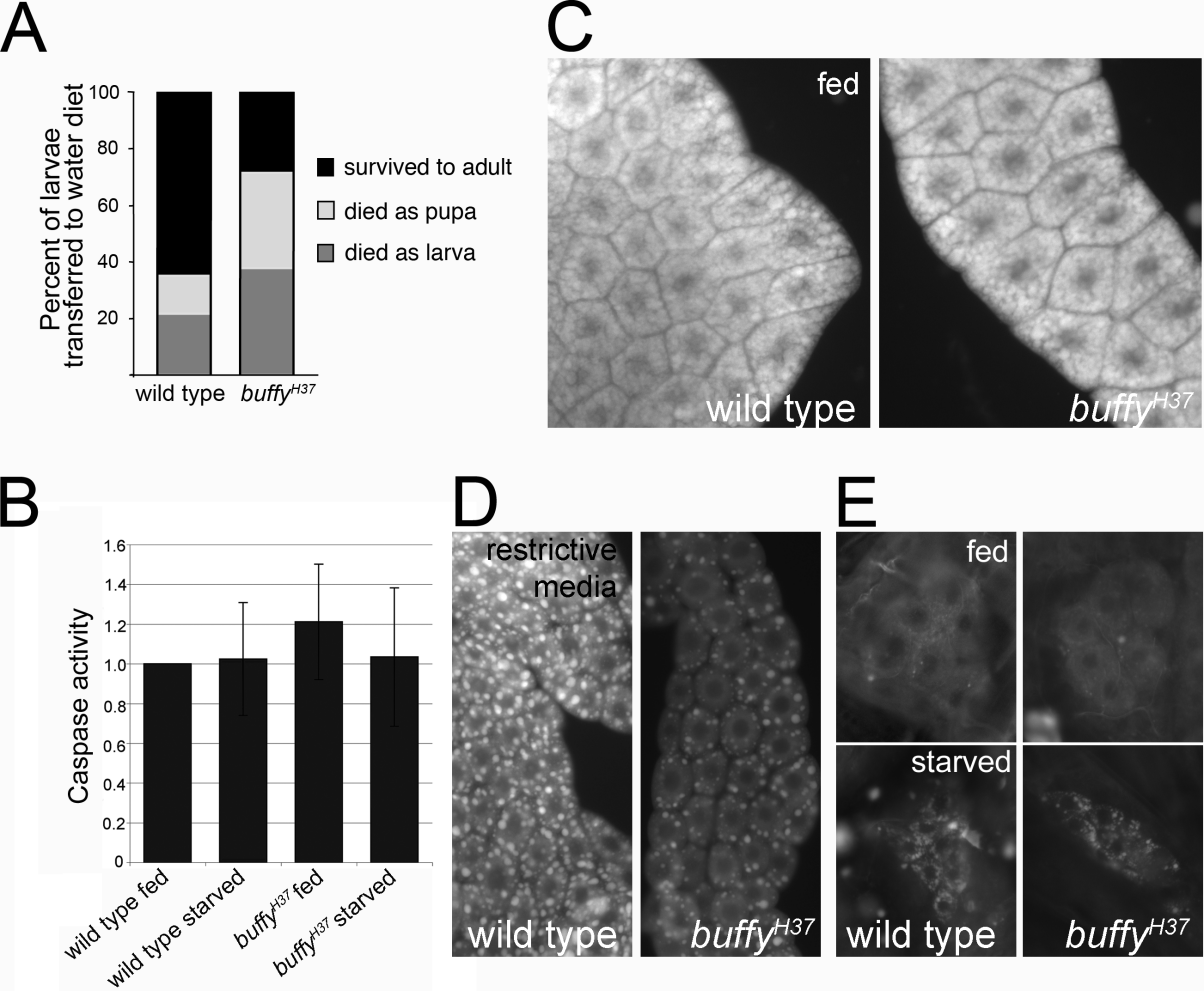
***buffy *mutants are sensitive to starvation, store less lipid and glycogen yet are capable of mobilizing fat stores**. **(A) ***buffy *mutants are twofold more sensitive to acute starvation conditions. Fed third instars (73 h after egg laying) were transferred to a water-only diet (*buffy^H37^*, n = 426; wild-type, n = 329). The graph indicates the percentage of transferred larvae that survived (dark bars: *buffy^H37^*, 29%; wild-type, 65%), that died as larvae (dark gray bars: *buffy^H37^*, 37%; wild-type, 22%) and that died as pupae (light gray bars: *buffy^H37^*, 34%; wild-type, 14%). **(B) **DEVDase activity of larval lysates for the indicated genotypes fed complete medium or after amino-acid starvation (three assays, each normalized to wild-type fed ± SEM). **(C) **Fat bodies from fed third instar larvae stain intensely with Nile Red. **(D) **Lipid droplets within *buffy *mutant fat body cells are smaller and stain less intensely compared to wild-type when third instar larvae were reared in restrictive media (20% cornmeal/yeast/agar food, 1.8% sucrose). Fat bodies stained with Nile Red. **(E) **Lipids accumulate in oenocytes when larvae are starved. Nile red stain of wild-type (left panels) and *buffy *mutant (right panels) oenocytes from fed larvae (top panels) or starved larvae (bottom panels).

Our study began by assessing the ability of *buffy *mutant larvae to survive acute nutrient starvation. When larvae are starved prior to 70 h after egg laying (AEL), they die within several days. Larvae that are starved beyond this time point pupate early, but undergo the normal developmental program of metamorphosis and produce viable, fertile adult flies [32]. We utilized this fact to test whether mutant larvae maintained normal energy stores and mobilized them when deprived of nutrients. *buffy *mutant and wild-type larvae, reared in nutrient rich conditions (grown without crowding and fed complete medium) were moved at 73 h AEL into vials containing water-moistened filter paper. For both genotypes, the majority of the larvae pupated. Animals deficient in *buffy *were twofold more sensitive to nutrient starvation: 65% of the starved wild-type larvae developed into flies, in comparison with 29% of the *buffy *mutant larvae (Figure 1A, dark bars). Most of this difference in survivorship was due to death during metamorphosis (Figure 1A, light gray bars).

The *Drosophila *Bcl-2 proteins play a role in stress-induced apoptosis [11-13]; therefore, we addressed the possibility of an apoptotic response to starvation. There was no evidence of caspase activation (Figure 1B) or terminal deoxynucleotidyltransferase dUTP nick-end labeling (TUNEL; data not shown) in *buffy *mutant or wild-type larvae. We conclude that protein starvation does not elicit an apoptotic response.

### *buffy *mutant larvae store less lipid and glycogen in the fat body and mobilize lipids upon starvation

The finding that Buffy is needed for survival following starvation-activated metamorphosis could indicate reduced lipid and glycogen storage. Lipids are stored in lipid storage droplets in the fat body that can be identified using the lipophilic stain, Nile Red. By the time larvae have reached the third instar stage, they have had over two days of continuous feeding with concomitant storage of lipids; therefore, their fat bodies stain intensely with Nile Red (Figure 1C). Several measurements were taken to compare lipid storage between *buffy *mutant and wild-type fat bodies: mean luminosity of Nile Red-stained fat bodies, quantification of Nile Red luminescence in larval lysates and quantification of the main constituent of fat stored in lipid droplets, triacylglyceride (TAG). We observed quite a bit of diversity in these measurements due to both natural disparities between animals as well as variation within each animal's fat body. Nevertheless, the trend in all three measurements was a reduction in the amount of lipid stored in *buffy *mutant larvae (Table 1).

**Table 1:** *buffy *mutant larvae maintain smaller steady-state lipid and glycogen stores

	Wild-type	*buffy^H37^*	*buffy^H37^/wt*
**Complete medium:**			
Nile Red fat body (mean luminosity)	62.4 ± 3.6 (3)	50.4 ± 2.5 (3)	0.81
Nile Red lysate (FU)	323 ± 47 (4)	260 ± 16 (4)	0.80
Triacylglyceride (μg TAG/μg protein)	0.56 ± 0.05 (4)	0.47 ± 0.04 (4)	0.85
Glycogen (ng glycogen/μg protein)	1.54 ± 0.16 (6)	1.30 ± 0.11 (5)	0.85
**20% medium:**			
Nile Red fat body (mean luminosity)	62.2 ± 15.5 (3)	46.3 ± 1.6 (3)	0.74
Number of lipid droplets/fat body cell	30.7 ± 5.3 (3)	20.7 ± 3.4 (3)	0.67
Perimeter of lipid droplets	38.9 ± 3.4 (3)	32.3 ± 2.2 (3)	0.83
**Complete medium + added sucrose:**			
Triacylglyceride (μg TAG/μg protein)	0.87 ± 0.07 (4)	0.74 ± 0.10 (4)	0.85

All experiments were performed on third instar larvae fed the indicated medium. All data are presented as averages ± SEM with number of biological replicates in parentheses. With the exception of mean luminosity of Nile Red fat body in complete medium (*P *= 0.05), all other differences are not significant (*P *> 0.1) as determined by Student's unpaired two-tailed t test. Nevertheless, the trend is similar across all measurements. For TAG assays, a representative of four biological replicates is presented. For technical reasons the mean luminosity cannot be compared across different media conditions (see Methods for experimental details).

When nutrients and sugar were reduced (20% cornmeal/yeast/agar food (CY), 1.8% sucrose; see Methods for media description), less lipid was stored in larval fat bodies, reflecting reduced fat intake and carbon availability. Because the fat bodies stained much less intensely with Nile Red, visualization of lipid-storage droplets was enabled (Figure 1D). Quantification of Nile Red-stained droplets demonstrated that larvae lacking *buffy *stored less lipid in fewer lipid droplets of smaller size in comparison to wild-type controls (Table 1). *buffy *knockdown larvae, in which ubiquitous expression of a hairpin RNA targeting *buffy *significantly reduced *buffy *expression, also displayed reduced Nile Red staining (see Additional file 1, Figure S1). Supplementation of the growth medium with excess sucrose (100% CY, 20% sucrose) led to an increase in TAG storage (TAG generated through sugar metabolism) for both mutant and wild-type larvae, although the *buffy *mutant still stored less TAG relative to wild-type (Table 1). We investigated whether continual breakdown of lipids contributed to the small reduction in fat storage in the *buffy *mutant. In fasting *Drosophila *larvae, fat that is released by the fat body is metabolized through β oxidation in specialized hepatocyte-like cells called oenocytes [33]. Oenocytes are clustered along the larval body wall and, in feeding animals, contain little to no fat; however, upon nutrient withdrawal, oenocytes from starving animals stain robustly with Nile Red as lipids are catabolized [33]. *buffy *does not influence the process of fat catabolism since oenocytes from *buffy *mutant larvae were indistinguishable from oenocytes in wild-type larvae, whether feeding or following starvation (Figure 1E).

Another stored source of energy for the larva is glycogen. The concentration of glycogen in *buffy *mutant larvae, grown in complete medium, followed the same trend as lipid concentration (Table 1). Taken together, these data demonstrate that larvae lacking *buffy *are able to generate and store lipid and glycogen and mobilize lipids upon starvation. However, *buffy *mutants maintain a reduced steady-state level of energy sources in the fat body, which likely contributes to the nutrient starvation phenotype.

### Larvae lacking *buffy *display metabolic changes similar to wild-type larvae following starvation, but have distinct metabolic characteristics in fed conditions

Energy metabolism also affects the ability of an animal to survive nutrient stress. When animals are subjected to nutrient stress, their metabolism shifts from favoring energy-requiring processes, such as protein synthesis and growth, to energy-generating processes such as glycolysis and oxidative phosphorylation. We investigated whether an inappropriate metabolic response to starvation also contributed to the inability of the *buffy *mutant to survive nutrient starvation.

**Figure 2:**
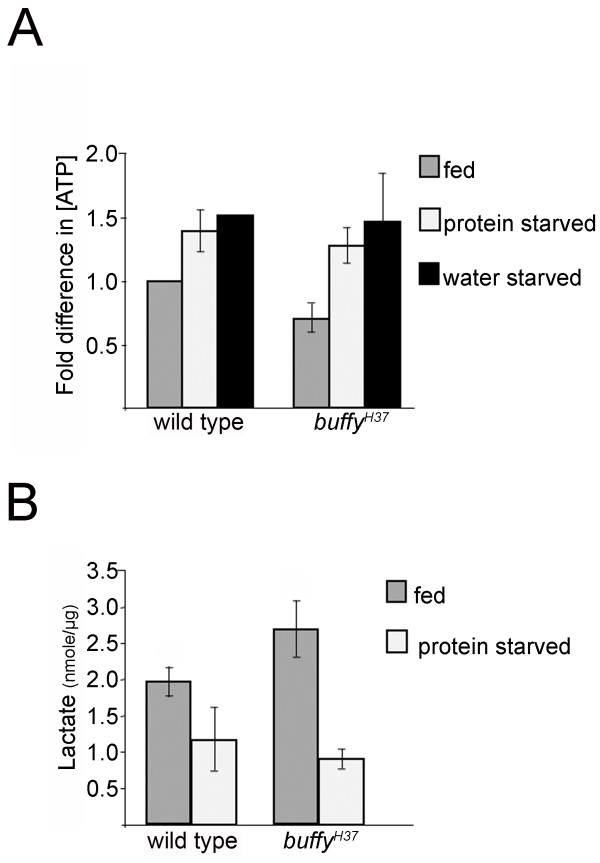
***buffy *mutants maintain lower ATP and higher lactate concentrations than wild-type larvae**. **(A) **ATP concentration in fed *buffy *mutant larvae is lower than wild-type. ATP concentration is expressed relative to wild-type fed. Mean ± SEM (n = 3 for fed and protein-starved experiments, n = 2 for water-starved experiment). Fed *buffy *ATP concentration was 48%, 82% and 83% of wild-type in the biological replicates. **(B) **Fed larvae lacking *buffy *have a higher concentration of lactate compared to wild-type larvae. Lactate concentration was determined for lysates from pools of 15 larvae. Mean ± SEM (wild-type fed, n = 5; *buffy^H37 ^*fed, n = 5; wild-type starved, n = 3; *buffy^H37 ^*starved, n = 4).

A very early response to acute starvation is activation of AMPK. Phosphorylated AMPK was detected within 30 minutes of starvation in both *buffy *mutant and wild-type larval extracts (data not shown). AMPK activates catabolic pathways that generate ATP while at the same time switching off ATP-consuming anabolic pathways. Reflecting AMPK activation, ATP concentrations increased in both mutant and wild-type larvae following starvation as expected (Figure 2A, light gray and black bars). This was not due to upregulation of glycolysis since the effect was the same whether the larvae were fed sucrose or water. Instead, this likely reflects reduced ATP consumption. The fact that ATP concentrations increased to roughly similar levels in *buffy *mutant and wild-type larvae demonstrates that *buffy *is not required for this metabolic response to starvation. But we noticed a curiosity in the control, fed larvae: fed *buffy *larvae maintained a reduced pool of ATP relative to wild-type (Figure 2A, dark gray bars).

Both glycolysis and the more efficient mechanism of oxidative phosphorylation contribute to ATP generation in the *Drosophila *larva. Because the ATP concentration was similar in starved *buffy *mutant and wild-type larvae, we expected that changes in the relative contributions of glycolysis and oxidative phosphorylation to energy generation following starvation were also similar. To examine this, the concentration of lactate was determined as an indication of glycolytic rate. Upon protein starvation, the lactate concentration dropped in both mutant and wild-type larvae, confirming that *buffy *mutant larvae were comparable to wild-type in their starvation response (Figure 2B, light gray bars). However, we noted a distinction between fed wild-type and *buffy *larvae: fed larvae lacking *buffy *maintained a higher steady-state concentration of lactate (Figure 2B, dark gray bars). It should be noted that the experiments presented in this section were performed on larval extracts in which the major component is fat body (roughly 80% of the animal's weight). Muscles were removed with the cuticle (no detergent was used during lysis) making the muscle contribution small.

We conclude that, with regard to energy generation and the parameters tested here, *buffy *mutant larvae responded normally to starvation. However, our experiments unexpectedly uncovered differences between fed mutant and wild-type larvae. At this stage of development, larvae feed virtually continuously in order to develop and grow adult structures and store enough nutrients to survive the non-feeding period of metamorphosis. Larvae lacking *buffy *maintain low ATP concentration, a high concentration of lactate, and smaller glycogen and lipid stores. All of these are consistent with an abnormal basal metabolism that places the animal closer to an energetic breakpoint, thus rendering the *buffy *mutant unusually sensitive to nutrient stress.

### Increased phosphorylated S6K, indicative of increased Tor signaling, is observed in *buffy *larvae

**Figure 3:**
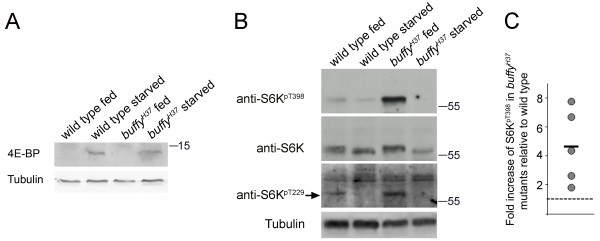
**Increased phosphorylated S6K is observed in fed *buffy *mutant larvae**. **(A) **A representative immunoblot demonstrating that 4E-binding protein (4E-BP) protein is detected in protein-starved wild-type and *buffy *larvae. **(B) **More phosphorylated S6K is detected in fed *buffy^H37 ^*mutant larval extracts relative to wild-type and target of rapamycin (Tor) signaling is downregulated by starvation in both genotypes. Data shown is from successive incubation with the indicated antibodies on the same western blot. **(C) **Graphical representation of fold increase of phosphorylated S6K in *buffy *mutants relative to wild-type (= 1, dashed line) for five separate biological experiments with the mean indicated by the horizontal bar.

We next investigated survival and growth signaling that mediates metabolic responses to nutrient status. Signaling through the PI3K/Tor pathway promotes growth when nutrients are present, but is quickly inactivated when nutrients are withdrawn [22,34-37]. In nutrient-rich conditions, PI3K signaling is active. Loss of signaling through PI3K results in Forkhead box O (FOXO) activation and subsequent transcription of FOXO targets, one of which is 4E-binding protein (4E-BP) [38,39]. 4E-BP protein was readily detected following protein starvation of *buffy *mutant larvae (Figure 3A), demonstrating that the loss of nutrients was properly relayed to stop growth signaling through the PI3K pathway.

Tor, a downstream effector of PI3K signaling, is a Ser-Thr kinase that positively regulates growth by promoting ribosome biogenesis, protein synthesis, and nutrient import while at the same time negatively regulating the catabolic process of autophagy. Following loss of a positive nutrient signal, Tor signaling is inactivated resulting in inhibition of energy-requiring processes such as protein synthesis [40]. Tor signaling was monitored through S6K phosphorylation as another indicator of the response to starvation. Reflecting the loss of the positive nutrient signal, the amount of phosphorylated S6K dropped in starved *buffy *mutant larvae (Figure 3B; see also Figure six). Intriguingly, more phosphorylated S6K was detected in fed *buffy *mutant larvae indicating that the basal level of signaling through Tor was high in the *buffy *mutant (Figure 3B, quantification of five independent experiments shown in Figure 3C). Tor phosphorylates S6K on T398 (equivalent to T389 in mammalian S6K) and subsequent phosphorylation of S6K on T238 (T229 in mammalian S6K) by 3-phosphoinositide-dependent kinase 1 (PDK1) is required for full activation of S6K [41,42]. Increased S6K T238-phosphorylation was also observed in *buffy *mutant larvae (Figure 3C). Furthermore, phosphorylated S6K (T398) was higher in fed *buffy *knockdown larvae relative to control larvae (see Additional file 2, Figure S2). Lastly, S6K phosphorylation in *buffy *mutant larvae was sensitive to *Tor *gene dosage (see Figure six). We conclude that larvae lacking *buffy *maintain hyperactive Tor signaling in their basal feeding state.

Clearly, the inability of the *buffy *mutant to survive nutrient withdrawal was not due to inappropriate signaling or metabolic responses to starvation. Instead, our study uncovered differences in basal energy storage and metabolism, as well as increased basal signaling through Tor that positively regulates growth.

### Lack of *buffy *results in precocious onset of autophagy following amino-acid withdrawal

Autophagy is a well-characterized response to starvation and is activated in the *Drosophila *larval fat body hours after larvae are protein starved [22,43]. This catabolic process involves the non-selective engulfment of cytoplasm, containing organelles and proteins, in double-membrane vesicles called autophagosomes [44,45]. Autophagosomes move to and fuse with a lysosome, forming an autolysosome, where breakdown of organelles and cytoplasmic proteins occurs to allow recycling of these critical building blocks when nutrients are unavailable. Since one of the roles played by Tor is to inhibit autophagy when nutrients are plentiful, we reasoned that *buffy *larvae with increased Tor signaling might fail to activate autophagy following protein withdrawal. Loss of autophagy could also contribute to starvation sensitivity.

**Figure 4:**
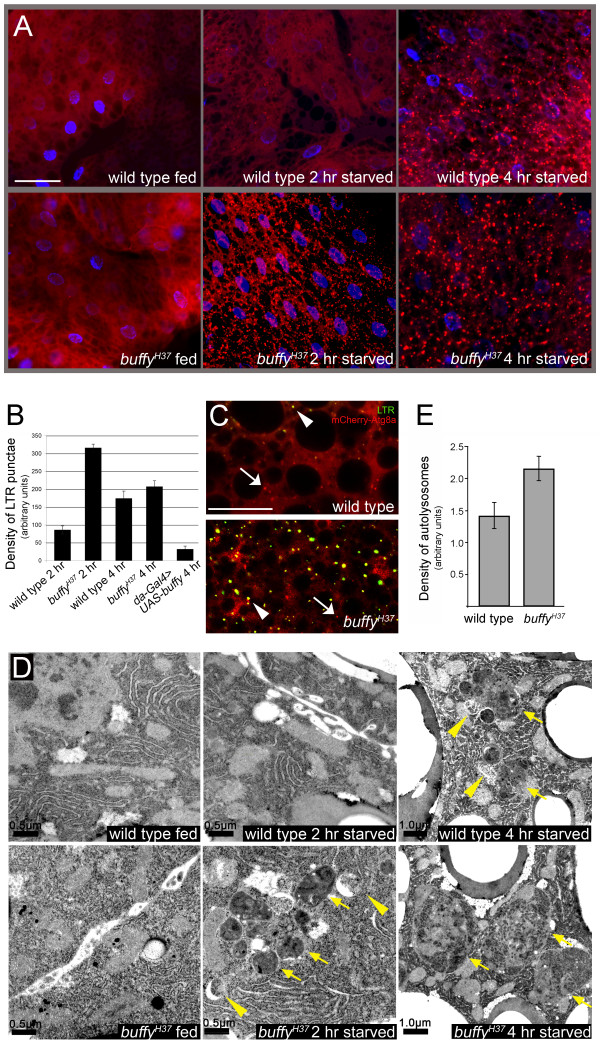
**Starvation-induced autophagy is initiated faster in the *buffy *mutant fat body in comparison to wild-type fat body**. **(A) **Timecourse showing LysoTracker Red (LTR) staining following 0, 2 and 4 h of amino-acid starvation. Blue represents Hoechst-stained nuclei (note that not all nuclei are accessible to Hoechst in live tissue). Robust LTR-positive staining is visible after 2 h of starvation in the *buffy *mutant fat body, whereas very few LTR-positive punctae are visible at this time point in wild-type. Scale bar = 47 μm. **(B) **Quantification of density of LTR punctae from 5 to 10 animals per genotype after 2 and 4 h of starvation. Mean ± SEM. **(C) **Fat bodies from 2 h-starved larvae expressing mCherry-Atg8a (red) and stained with LTR (green). Both autophagosomes (arrows) and early autolysosomes (arrowheads) were labeled with mCherry-Atg8a. Scale bar = 38 μm. **(D) **Transmission electron microscopy (TEM) images of fat bodies from larvae starved for 0, 2 and 4 h demonstrating detection of autophagosomes (yellow arrowheads) and autolysosomes (yellow arrows). Scale bars are as indicated. **(E) **Quantification of autolysosomes in TEM images from 4 h-starved larval fat bodies.

The initial assessment of protein starvation-induced autophagy utilized LysoTracker Red (LTR), a stain that marks acidic vesicles such as autolysosomes and lysosomes. Fat bodies from feeding larvae have very few lysosomes, and thus very little LTR staining, but upon transfer to protein-free media, (20% sucrose or water only), abundant autolysosomes are visualized as punctate LTR staining ([22,43] and Figure 4A). In wild-type larvae, a small number of LTR puncta are visible after 2 h of amino-acid withdrawal and robust LTR staining is observed after 4 h ([22,43] and Figure 4A, B and see Additional file 3, Figure S3). *buffy *null animals were competent in initiating autophagy; however, the autophagic response was faster with strong LTR staining visible after 2 h of protein starvation (Figure 4A, B, and Additional file 3, Figure S3). Similarly robust LTR staining after 2 h of starvation was also observed in *buffy *knockdown larvae (Additional file 1, Figure S1). Protein starvation-induced autophagy in the larval fat body is transient, dropping after 12 to 24 h [22,43] and LTR staining in the starved *buffy *mutant followed a similar timecourse (data not shown).

Because LTR is not specific for autolysosomes, other methods of marking autophagic structures were employed to corroborate the LTR data. LC3-green fluorescent protein (GFP) and mCherry-ATG8 identify autophagosomes and early autolysosomes [43,46]. Both markers confirmed that larvae lacking *buffy *activate the autophagic pathway faster than wild-type larvae (Figure 4C, and Additional file 3, Figure S3). Transmission electron microscopy (TEM) demonstrated that no autophagic structures were observed in fat bodies of mutant and wild-type larvae grown in complete medium and that lysosomes were rarely seen (Figure 4D). After 2 h of starvation, micrographs revealed many autolysosomes in the *buffy *mutant fat body whereas an autolysosome was rarely observed in the wild-type fat body (Figure 4D, quantified in Figure 4E).

### Buffy does not directly inhibit formation and maturation of autophagic vesicles

Faster autophagy in response to starvation was the opposite of the expected outcome of high Tor signaling. A possible explanation is that Buffy negatively regulates autophagy, downstream of Tor signaling, to delay onset following starvation. 'AuTophaGy' related 1 (ATG1) is a Ser-Thr kinase that controls the autophagic machinery in response to Tor signaling and mutation of *Atg1 *blocked starvation-induced autophagy in *Drosophila *larvae [22,47]. We generated the *Atg1 buffy *double mutant. No LTR staining was observed in the fat bodies of fed or starved double mutant larvae (data not shown), indicating that loss of *buffy *was unable to derepress autophagy downstream of *Tor *and *Atg1*.

**Figure 5:**
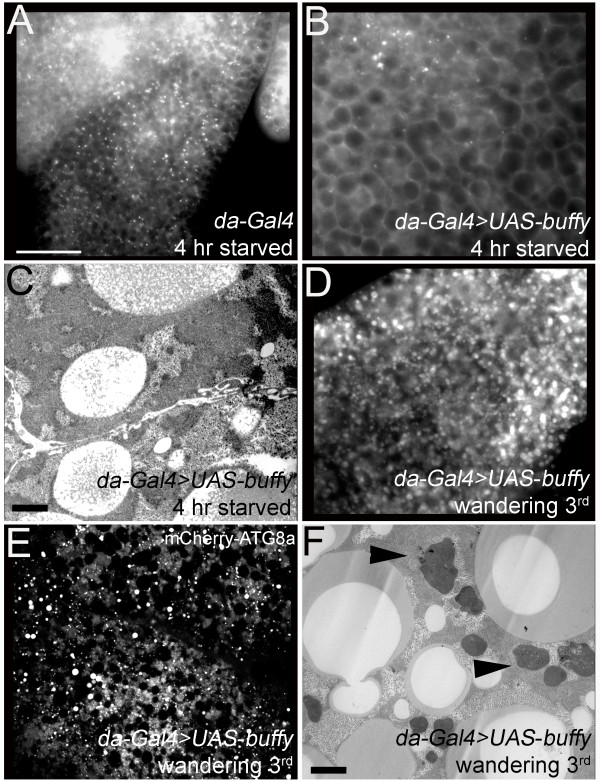
**Ectopic Buffy inhibits autophagy following 4 h of amino-acid starvation but does not block programmed autophagy**. **(A, B) **LTR staining of fat bodies from 4 h-starved larvae expressing *da-Gal4 *(A) or *da-Gal4; UAS-buffy *(B). **(C) **TEM of fat body from *da-Gal4; UAS-buffy *after 4 h of starvation with no visible autophagic structures. **(D-F) **Autophagic vesicles are clearly visible in *da-Gal4; UAS-buffy *fat body from late third instar (wandering) larvae demonstrated by LTR staining (D), mCherry-Atg8a (E), and TEM (F); arrowheads point to autolysosomes). Scale bar = 47 μm (A, B, D, E) and 2.0 μm (C, F).

We investigated whether ectopic Buffy inhibited starvation-induced autophagy. Indeed, overexpression of Buffy in the *Drosophila *larva delayed the autophagic response to starvation. After 4 h of starvation, LTR staining of fat bodies from animals with ectopic Buffy was dramatically reduced (Figure 5A, B; quantification shown in Figure 4B). Due to non-uniform expression of *Gal4 *in fat body, some regions had no LTR staining and other regions had a small number of LTR puncta; however robust LTR staining was observed throughout the control fat body at this timepoint. LTR staining was confirmed by EM analysis (Figure 5C, compare to Figure 4D). Ectopic Buffy, however, did not simply block the autophagic response, but rather delayed the response: extensive autophagy was apparent in fat bodies with ectopic Buffy after an extended period of protein starvation (data not shown).

Prior to larval pupation, the fat body undergoes autophagy in response to hormonal changes [43]. Ectopic Buffy did not inhibit programmed autophagy, as demonstrated by LTR staining, mCherry-ATG8a and EM analysis (Figure 5D-F). These data, together with the *Atg1 *genetic interaction data, demonstrate that Buffy does not inhibit formation and maturation of autophagic vesicles, but instead blocks the induction signal that is specific to the protein-starvation response upstream of the autophagic machinery.

Thus far, our study demonstrated that larvae lacking *buffy *were unable to survive acute nutrient starvation. Loss of *buffy *resulted in increased basal Tor signaling and altered energy storage and metabolism, but did not change the way the animal responded to protein starvation, with the exception of faster autophagy. This last phenotype was the opposite expected for increased Tor signaling (Tor inhibits autophagy and thus should slow the response). The next series of experiments were designed to understand the consequence of increased Tor signaling in the *buffy *mutant.

### *buffy *null animals do not rely on increased Tor signaling for viability

**Figure 6:**
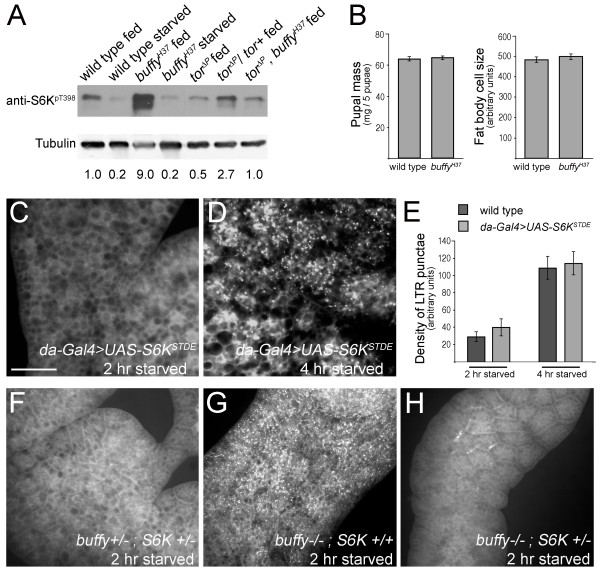
**Increased phosphorylated S6K in the absence of *buffy *poises the animal for starvation-induced autophagy and is target of rapamycin (Tor) dependent**. **(A) **Increased phosphorylated S6K is no longer detected in the *buffy *mutant when Tor signaling is blocked. Immunoblot of lysates from fed and 2 h-starved larvae of the indicated genotypes (all are homozygous with the exception of the heterozygous *tor*^Δ*P*^*/tor+ *control) using the indicated phosphospecific S6K antibody. The ratio of phosphorylated S6K to tubulin loading control is indicated below each lane, relative to wild-type fed. **(B) **Increased phosphorylated S6K in the *buffy *mutant does not promote growth. Left panel: the average mass of five staged pupae is similar for *buffy *mutant and wild-type animals. Mean ± SEM; n = 20 groups of five for both wild-type and *buffy *mutant. Right panel: the average fat body cell size is similar for *buffy *mutant and wild-type animals. Mean ± SEM; n = 97 fat body cells from 4 animals for *buffy *mutant and 119 fat body cells from 5 animals for wild-type. **(C, D) **LTR-positive punctae are not visible in fat bodies from *da-Gal4; UAS-S6K^STDE ^*larvae after 2 h of amino-acid starvation, but are after 4 h of amino-acid starvation (compare with (A)). **(E) **Quantification of density of LTR-positive punctae from 15 animals per indicated genotype after 2 and 4 h of starvation. Mean ± SEM. **(F-H) **Removal of a genomic copy of S6K reverts the precocious autophagy phenotype of the *buffy *mutant, as demonstrated by LTR staining of fat bodies from larvae starved for 2 h. (F) *buffy^H37^*/+; *S6K^l-1^*/+ fat body with wild-type autophagy. (G) *buffy^H37 ^*homozygous mutant fat body with robust autophagic response. (H) *buffy^H37^*/*buffy^H37^*; *S6K^l-1^*/+ fat body demonstrating reversion of autophagic phenotype to wild-type. Images are representative of seven to ten animals per genotype.

We hypothesized that *buffy *larvae might rely on excess Tor signaling for viability and that this would render them sensitive to severe reduction in Tor signaling. To test this, we generated animals that were *buffy *null and *Tor *null double mutants. No genetic interaction was observed; *buffy Tor *double mutant larvae hatched in equal numbers and grew at the same rate as *Tor *single mutant larvae (data not shown). We confirmed that phosphorylated S6K was no longer increased in larvae that were doubly deficient for *buffy *and *Tor *(Figure 6A). Clearly, increased phosphorylated S6K is not required for survival of larvae lacking *buffy*. Further interpretation of this experiment is confounded by two points: firstly, even though phosphorylated S6K is reduced, it is still detectable and secondly, larvae of the *Tor *null mutant have a severely altered metabolism and maintain a phenotype of constitutive starvation in which autophagy is activated and nutrient intake is drastically reduced (due to lack of a positive input into insulin signaling by Tor [48,49]). An expected consequence of increased Tor signaling is growth [50-54]. We did not observe any increase in cell size in *buffy *mutant fat body, the *buffy *mutant animals grew at normal rate [11], fat body cells were of the normal size distribution and no difference in pupal mass was observed (Figure 6B). Furthermore, if a consequence of Tor signaling were increased growth (and increased rate of energy consumption), precocious autophagy could occur because starvation causes Tor signaling to be shut off more rapidly in the *buffy *mutant than wild-type controls. However, this is not the case as the level of phosphorylated S6K in 2 h-starved *buffy *mutant larvae was equal to or higher than starved control larvae in several experiments (Figure 6A; ratio of phosphorylated S6K in *buffy *mutant relative to wild-type in four separate experiments: 1.1, 4.4, 4.0, 1.6).

### Increased phosphorylated S6K in the *buffy *mutant poises the animal for starvation-induced autophagy

We investigated the possibility that activated S6K was sufficient to induce precocious starvation-induced autophagy in wild-type larvae. Ectopic expression of activated S6K was not sufficient to initiate autophagy in fat bodies of fed larvae [22]. Ectopic activated S6K in the wild-type fat body also did not affect the timing of initiation of protein starvation-induced autophagy or the density of LTR-positive puncta (Figure 6C-E).

To investigate whether the increased phosphorylated S6K was necessary for precocious starvation-induced autophagy in the absence of *buffy*, we wished to reduce the intracellular pool of phosphorylated S6K in *buffy *null larvae. Removing one genomic copy of S6K, by crossing in a null S6K allele, has been successfully used in other studies to drop active S6K levels (for example, see [52,55-57]). Heterozygous control larvae (*buffy^H37^*/*buffy^+^*; *s6k^l-1^*/*s6k^+^*) had little to no LTR staining after 2 h of protein starvation (Figure 6F), whereas robust LTR staining was observed in the *buffy *mutant fat body (Figure 6G). In contrast, no LTR-positive puncta were visible in the experimental *buffy *mutant larvae with only one copy of wild-type S6K (*buffy^H37^*/*buffy^H37^*; *s6k^l-1^*/*s6k^+^*; Figure 6H). These results demonstrate that when larvae lack *buffy*, an increased pool of phosphorylated S6K is sufficient to induce precocious autophagy in fat body cells upon protein starvation. When Buffy protein is present, increased phosphorylated S6K is not sufficient for the precocious autophagic response to nutrient withdrawal.

The next series of experiments made use of starvation-induced autophagy as an indicator of Tor signaling in order to investigate the interaction between nutrient stress and Tor.

### Nutrient withdrawal activates autophagy in the *buffy *mutant faster than rapamycin-mediated inhibition of Tor in contrast to wild-type

Because reduction in S6K abolished precocious initiation of starvation-induced autophagy in the *buffy *mutant, we wished to downregulate Tor signaling in order to directly test the contribution of Tor to the phenotype. Ectopic expression of a dominant-negative truncated Tor protein in single cells within the fat body is sufficient to activate autophagy, even in fed animals [22]. Downregulation of Tor through expression of the dominant-negative protein also induced autophagy in the *buffy *mutant, preventing experimental determination of the timing of autophagy (see Additional file 4, Figure S4). LTR staining in cells expressing dominant-negative Tor was indistinguishable from cells with normal Tor signaling in the *buffy *mutant fat body following protein starvation (Additional file 4, Figure S4).

**Figure 7:**
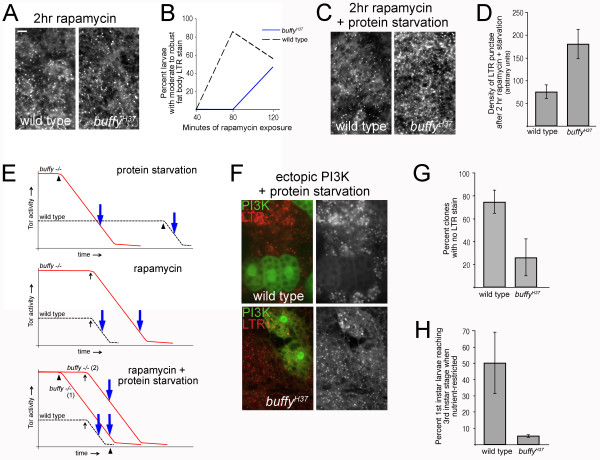
**Target of rapamycin (Tor) inhibition and protein starvation are both required for precocious autophagy in *buffy *mutant fat body**. **(A) **After 2 h of rapamycin treatment, LTR staining of wild-type and *buffy *fat bodies are roughly equivalent. Scale bar = 11.8 μm and is the same for all images. **(B) **Quantification of the percentage of larvae with moderate to robust fat body LTR stain after the indicated minutes of rapamycin exposure (number of larvae: 40 minutes: wild-type and *buffy^H37 ^*n = 4; 80 minutes: wild-type n = 7, *buffy^H37 ^*n = 5; 120 minutes: wild-type n = 16, *buffy^H37 ^*n = 15). **(C) **The addition of 2 h of protein starvation to rapamycin treatment induces a robust LTR-positive response in the *buffy *mutant. **(D) **Quantification of the density of LTR-positive punctae in fat bodies from larvae treated with rapamycin and protein starved for 2 h (number of larvae: wild-type, n = 7; *buffy^H37^*, n = 5). **(E) **Schematics to describe data from (A-D). In the top panel depicting protein starvation, Tor activity drops from a greater level in the *buffy *mutant relative to wild-type following the timepoint (arrowheads) at which nutrient stress is sensed. Autophagy is initiated later in wild-type than in the *buffy *mutant (blue arrows). In the middle panel depicting rapamycin feeding, rapamycin inhibition of Tor begins at the same time (vertical black arrow) in both genetic backgrounds, but because there is more Tor activity in the *buffy *mutant, autophagy initiation is delayed (blue arrows). In the bottom panel depicting rapamycin plus protein starvation, two scenarios are presented. In (1), the nutrient stress input (arrowhead) is faster than rapamycin inhibition (vertical black arrow) in inactivating Tor activity. In (2), the nutrient stress input (arrowhead on Time axis) activates autophagy (blue arrow) before Tor is fully inhibited. Only the wild-type curve for rapamycin is shown since autophagy is activated faster than starvation in this condition. In this graph, autophagy is quicker in wild-type relative to the *buffy *mutant, but it could also be slower. **(F) **Ectopic phosphoinositide 3-kinase (PI3K) is inefficient at inhibiting starvation-induced autophagy when *buffy *is absent. Cells with ectopic PI3K signaling are indicated by GFP. For each genotype a representative image of the major LTR pattern observed is presented. The left panel shows the merge of GFP (green) and LTR (red) and the right panel shows the LTR pattern alone. Genotypes: *y w hsFLP*; *buffy^H37^/+; actin > CD4 > Gal4*, *UAS-GFP/UAS-dp110 *and *y w hsFLP*; *buffy^H37^/buffy^H37^; actin > CD4 > Gal4*, *UAS-GFP/UAS-dp110*. **(G) **Quantification of the percentage of clones with ectopic PI3K signaling that had no LTR-positive punctae. Clones (control, n = 123; *buffy^H37^*, n = 66) were from ten control larvae and seven *buffy^H37 ^*larvae. In addition to the percentage of clones with no LTR-positive punctae, the number of clones with reduced number or intensity of LTR punctae was also quantified: control, 19%; *buffy^H37^*, 22%. The remaining clones (control, 6%; *buffy^H37^*, 52%) had an LTR response equivalent to neighboring cells. **(H) ***buffy *mutant larvae are unable to adapt to nutrient restriction. First instar larvae were transferred to nutrient-restriction medium (20% cornmeal/yeast/agar food (CY), 9% sucrose) and the percentage of these larvae that developed into third instar larvae after 6 days is presented (number of larvae: wild-type, n = 132; *buffy^H37 ^*n = 231). Mean ± SEM.

To more directly investigate the timing of autophagy initiation in the *buffy *mutant, we employed the drug rapamycin to temporally inhibit Tor signaling. After 2 h of rapamycin feeding, moderate to robust LTR staining was already visible in wild-type larval fat bodies (Figure 7A), demonstrating that direct inhibition of Tor signaling activates autophagy faster than nutrient deprivation in wild-type larvae. LTR-stained fat bodies from numerous individuals were examined at time points earlier than 2 h and classified as either 'zero to minor response' or 'moderate to robust response' to rapamycin. No LTR staining was visible in fat bodies after 40 minutes of rapamycin exposure, but after 80 minutes, 'moderate to robust' staining was evident in over 80% of wild-type fat bodies (Figure 7B). Unlike wild-type and in striking contrast to nutrient starvation, rapamycin-fed *buffy *mutant animals did not initiate autophagy faster than wild-type, but were instead slower: fat bodies from *buffy *mutant larvae displayed 'zero to minor' LTR staining even after 80 minutes of rapamycin feeding (Figure 7B). By 2 h of rapamycin exposure, LTR staining was fairly equivalent in both genetic backgrounds and declined in later time points (data not shown). The requirement for longer rapamycin exposure to initiate autophagy in the *buffy *mutant is a likely consequence of the observed higher basal Tor signaling. This is in contrast to the observation that starvation initiated autophagy faster in the *buffy *mutant and high basal Tor signaling was required for this phenotype. To confirm that nutrient withdrawal is more efficient at activating autophagy in the *buffy *mutant in comparison to rapamycin treatment, larvae were fed a combined rapamycin/protein-starvation media for 2 h. LTR stained fat bodies from wild-type larvae fed this media were similar to rapamycin-fed wild-type larvae (Figure 7C). Fat bodies from the *buffy *mutant, however, were more intensely stained with LTR after 2 h of rapamycin/protein-starvation treatment (Figure 7C). The increased autophagic response in the *buffy *mutant was confirmed by quantification of LTR-positive punctae (Figure 7D). To sum this data up (Figure 7E), rapamycin induced autophagy within 80 minutes in wild-type larvae, but protein-starvation took roughly three times longer to induce autophagy (4 h). In contrast, autophagy in the *buffy *mutant in response to protein starvation was faster than wild-type (2 h), even though rapamycin-induced autophagy was slower (2 h). Although it is formally possible that the nutrient-withdrawal signal inactivates Tor much faster than rapamycin in the *buffy *mutant (this possibility is not supported by the western blot analysis of phosphorylated S6K in starved larvae; Figures 3B and 6A), a simpler explanation is that the loss of the positive nutrient signal has an input into autophagy that is not Tor dependent.

### Lack of *buffy *is permissive for autophagy even when PI3K signaling is artificially maintained

To further test how nutrient withdrawal initiates autophagy in the *buffy *mutant, we investigated the effect of enforcing Tor signaling through ectopic expression of class I PI3K. Ectopic PI3K signaling overrides loss of a positive nutrient signal and suppresses starvation-induced autophagy [22,43]. Fat body cell clones were generated that express ectopic *dp110*, the catalytic subunit of class I PI3K, to activate PI3K signaling in larvae prior to protein starvation [22,43,58]. Cells with ectopic *dp110 *in both *buffy *mutant and wild-type fat bodies grew larger than neighboring cells as is expected for ectopic activation of PI3K signaling (data not shown). As shown previously, LTR-positive punctae in response to starvation were not visible in wild-type fat body cells with ectopic PI3K signaling (Figure 7F). In contrast, the majority of PI3K-expressing cell clones in starved *buffy *mutant fat body contained LTR-positive punctae (Figure 7F; quantified in Figure 7G). Several conclusions can be made from this experiment. First, Tor is known to suppress autophagy independently of S6K and this mechanism is ineffective in starved *buffy *mutant larvae. Second, the inability of rapamycin to phenocopy protein starvation in the *buffy *mutant is not due to a molecule competing with rapamycin for Tor binding as has been shown for phosphatidic acid [59]. Third, as suggested by the western blot analysis and rapamycin experiments and confirmed in this experiment, the protein-starvation input into autophagy in *buffy *larvae is either downstream of Tor or in a separate pathway from Tor. The finding that precocious initiation of starvation-induced autophagy requires augmented phosphorylated S6K, is strong evidence that S6K or a molecule downstream of S6K transmits the nutrient-withdrawal signal.

### *buffy *is required for adaptation to nutrient restriction

Taken together, the data presented thus far indicate that *buffy *is required for maintaining normal basal energy metabolic homeostasis and normal Tor signaling. This study was initiated by an observation that *buffy *mutants are sensitive to poor growing conditions. The findings of inappropriate energy metabolism, Tor signaling and reduced energy storage all could contribute to this. Because the acute starvation experiment reported in Figure 1A is likely influenced by nutrient storage, we designed a second assay to test survival following nutrient stress in which energy storage was expected to have less of an impact. The assay involved growing larvae from first instar on food that contained a reduced concentration of nutrients (20% CY, 9% sucrose). Fed this nutrient-restriction diet, wild-type larvae develop and molt albeit with a reduced growth rate and minimal mass accumulation. After 6 days of feeding on the reduced nutrient medium, half of the wild-type larvae reached the third instar developmental stage (Figure 7H). In stark contrast, larvae lacking *buffy *were unable to metabolically adapt to the nutrient stress and only 5% of the *buffy *mutant first instar larvae developed into third instar larvae (Figure 7H). Instead, despite continual foraging, *buffy *mutant larvae remained small without noticeable growth or development: a phenotype similar to Tor-deficient or protein-starved larvae [60,61]. Thus, even in the experimental setting in which utilization of stored energy sources is not required, *buffy *mutant animals were sensitive to nutrient stress. We conclude that *buffy *is required for either the attenuation of metabolic activity in response to nutrient stress or to maintain the basal metabolic state that is a requisite for adaptation.

**Figure 8:**
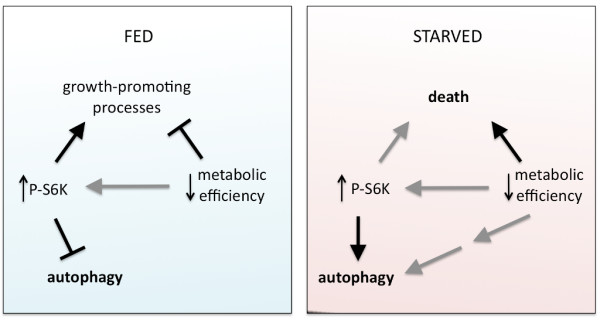
**In the *buffy *mutant, increased target of rapamycin (Tor) signaling and altered basal energetics, hypothesized to be due to reduced metabolic efficiency, are both required for the precocious autophagy phenotype and collaborate in the inability to survive nutrient stress**. Increased Tor signaling in fed animals fuels energy-intensive, growth-promoting processes. This is balanced by the altered energy metabolism as demonstrated by reduced lipid and glycogen storage and lack of overgrowth phenotypes. It is possible that the increase in Tor signaling is due to altered metabolism or that the two are independent of each other. However, the altered metabolism cannot entirely be due to increased Tor signaling because ectopic expression of activated S6K does not recapitulate precocious autophagy in a wild-type background. Upon starvation, loss of Tor signaling releases inhibition of autophagy while phosphorylated S6K promotes autophagy. Our data demonstrates that a second Tor-independent, nutrient-responsive input is required for precocious autophagy. This second input is hypothesized to arise from the altered basal energetics uniquely found in *buffy *mutants.

Putting the metabolic experiments together with phosphorylated S6K, we propose that Buffy plays a role in maintaining optimal metabolic conditions and adapting metabolism to nutrient stress. Concomitant with this, Tor signaling, as evidenced by phosphorylated S6K, is increased in *buffy *mutant larvae. Although this affects precocious starvation-induced autophagy, it does not appear to be the only input into precocious autophagy as a second nutrient-withdrawal input is required. Our experiments suggest that the second input is downstream of Tor or Tor independent and is related to the altered energy homeostasis in the *buffy *mutant (Figure 8).

## Discussion

All animals in their natural habitats are faced with periods of reduced nutrient availability. Our study demonstrated that the *bcl-2 *gene, *buffy*, is required for normal larval responses to nutrient stress. This could not be attributed to a role for *buffy *in sensing nutrient starvation and activating normal starvation responses. Instead, larvae lacking *buffy *displayed characteristics of altered energy metabolism and increased growth signaling through Tor, as demonstrated by increased phosphorylated S6K. Our study did not address whether the increased Tor signaling is a cause or result of the energy metabolism of the *buffy *mutant. It is conceivable that upregulation of Tor signaling results in increased energy consumption to promote growth. However, we did not observe that the increased phosphorylated S6K was correlated with increased growth in the *buffy *mutant, suggesting that the Tor signaling was balanced by the altered energy metabolism in the mutant. Taking into account the current understanding of Bcl-2 proteins (discussed briefly below), we postulate that Buffy is required to maintain energy homeostasis at a set point that is optimal for both growth and starvation responses. Loss of *buffy *results in a change of this homeostatic set point that may directly or indirectly upregulate growth signaling and that places the animal closer to a metabolic cliff in terms of its ability to survive nutrient stress.

In investigating starvation responses in the *buffy *mutant, we observed that fat body autophagy was initiated faster in *buffy *mutant larvae. Although Tor signaling normally inhibits autophagy, the high level of phosphorylated S6K maintained by the mutant was required for precocious starvation-induced autophagy. Since autophagy is a mechanism to recycle essential building blocks when nutrients in the environment are scarce, we investigated whether reduced energy storage was correlated with precocious autophagy. Wild-type animals, with normal Tor signaling, provided with 20% of the normal nutrients (20% CY, 1.8% sucrose) were autophagic after 2 h of starvation (unpublished observations, JPM and CBB). But this nutrient-restriction diet resulted in a much greater reduction in stored nutrients in the fat body than the 15% reduction in lipid storage observed in the *buffy *mutant (compare Figure 1C and 1D). In addition, excess growth signaling by ectopic activation of Tor signaling in wild-type larvae, was not sufficient to induce precocious autophagy (Figure 6). We propose that it is the unique combination of an altered metabolism, increased Tor signaling in larvae lacking *buffy *that renders the animal more sensitive to nutrient stress and results in precocious autophagy.

Energy sensing has been linked to autophagy initiation in mammals. ULK1 (mammalian ATG1) function is regulated by both Tor and AMPK. In the simplest current thinking, nutrient deprivation both inactivates Tor and activates AMPK to phosphorylate and activate ULK1 to initiate autophagy [62,63]. In *Drosophila*, the complex of ATG1/ATG13 is regulated by Tor [46] and AMPK is required for starvation-induced autophagy [64], suggesting that regulation of autophagy initiation by phosphorylation is similar in fruit flies. In larvae lacking *buffy*, decreased cellular energy (discussed further below) might more efficiently activate ATG1/ATG13, possibly mediated through AMPK. This model does not take into account that precocious autophagy in the *buffy *mutant required phosphorylated S6K. There is conflicting data as to the role of S6K in autophagy. Because inhibition of Tor induces autophagy, phosphorylation of S6K is inversely correlated with autophagy. However, S6K has been shown to be required for starvation-induced autophagy in *Drosophila *[22], and plays a positive role in autophagic induction in mammals [65,66]. Faster autophagy in the *buffy *mutant may reflect a positive signaling role for S6K in autophagy initiation that contributes to this phenotype. Indeed it is intriguing to postulate that a metabolic signal from loss of the positive nutrient signal is transmitted through phosphorylated S6K in all animals, and that augmented phosphorylated S6K merely potentiates this signal in the *buffy *mutant.

The metabolism phenotypes observed in the *buffy *mutant larvae (smaller energy stores in the fat body, increased glucose utilization inferred from less glycogen storage, a reduced pool of ATP and increased lactate) are most simply explained by a shift in the balance of glycolysis to oxidative phosphorylation toward glycolysis. Glycolysis is less efficient at generating ATP and increased glycolysis generates excessive pyruvate that is converted to lactate. To maintain glycolysis at a higher rate, a higher percentage of ingested glucose and lipids must be shuttled into glycolysis at the expense of storage in the fat body. Animals that rely more on glycolysis for energy generation would certainly be more sensitive to nutrient restriction. This hypothesis is supported by recent evidence that oxygen consumption and cellular ATP levels were reduced, while glycolysis was increased, in Bcl-2-associated X protein (BAX)-deficient cells [67]. Two recent studies on Bcl-x_L _also support direct regulation of oxidative phosphorylation: one demonstrated that Bcl-x_L _controls the levels of the metabolite acetyl coenzyme A (acetyl-CoA) [68] and the other proposed that neuronal Bcl-x_L _directly regulates the efficiency of ATP synthesis by the F_1_F_0 _ATP synthase complex [69]. Consistent with less efficient oxidative phosphorylation, *buffy *mutant larvae are sensitive to the reactive oxygen species (ROS) generator, paraquat, and have a twofold increase in ROS (JPM and CBB, unpublished observations). Increased ROS has also been reported to result from enforced Tor signaling in *Drosophila *[54]. Intriguingly, ROS has been proposed to affect S6K phosphorylation [70].

Bcl-2 proteins govern permeabilization of the mitochondrial outer membrane that leads to loss of mitochondrial energy production and release of apoptogenic factors such as cytochrome *c*. Buried within the vast quantity of publications investigating Bcl-2 proteins are studies that support a role for some of the Bcl-2 proteins in mitochondrial energetics (reviewed in [71]), generally with a focus on ectopic expression of Bcl-2 proteins and effects on metabolism with regard to apoptosis. Many studies have shown an interaction between Bcl-2 proteins and the voltage-dependent anion channel (VDAC) that regulates movement of metabolites between the mitochondria and the cytosol [72]. Although this interaction is not required for mitochondrial-dependent cell death [73], it may be that Bcl-2 proteins modulate mitochondrial energetics through VDAC. One of the metabolites whose uptake is facilitated by VDAC is Ca^2+^. Intracellular Ca^2+ ^signaling is regulated by the ER and Bcl-2 proteins influence ER calcium content through modulation of the inositol triphosphate receptor (IP3R) and the sarcoplasmic/endoplasmic reticulum calcium ATPase (SERCA) [74,75]. Uptake of Ca^2+ ^released by the ER can stimulate mitochondrial energy metabolism through several targets [7]. Ectopic Buffy decorates both the mitochondria and the ER in various cell types [16,17], leaving open the possibility that Buffy has a functional role in ER-mitochondria Ca^2+ ^signaling. Additionally, Bcl-2 proteins play a role in mitochondrial morphogenesis, both in the fragmentation observed upon apoptosis induction [76] and in healthy cells [77]. Mitochondria in *Drosophila *also fragment prior to cell death [14,78,79]. We observed that *buffy *mutant fat body had a higher density of mitochondria that were in general smaller and less 'snake like' (JPM and CBB, unpublished observations). However, *buffy *mutant animals did not have more mitochondria since no increase in mitochondrial genomes was observed in larval or fat body extracts (unpublished observations, JPM and CBB).

## Conclusions

This study has demonstrated that *Drosophila *larvae lacking the *bcl-2 *gene, *buffy*, are sensitive to nutrient restriction and starvation. *buffy *mutant larvae have unusual basal characteristics: increased Tor signaling, reduced energy source storage, reduced ATP levels and increased lactate levels. Our data provides evidence that, in the normal animal, Buffy maintains basal energy homeostasis to enable appropriate responses to nutrient stress. Future studies will determine how Buffy influences basal energy metabolism and clarify the relationship between energy metabolism and S6K regulation. The recent reports demonstrating that Bcl-x_L _regulates metabolic efficiency in neurons [69] and that Bax promotes bioenergetics in HCT-116 cells and primary hepatocytes [67] support the hypothesis that some Bcl-2 proteins have a non-apoptotic role to produce resistance to stressors by maintaining mitochondrial energetics. Our data adds to these reports, and is unique because it investigates the effect on organismal health of loss of a *bcl-2 *gene and provides evidence for crosstalk with Tor signaling. It is important to note that the *Drosophila *Bcl-2 proteins are *bona fide *Bcl-2 proteins containing BH1-4 domains and a C-terminal transmembrane domain, have the ability to bind other Bcl-2 proteins and can substitute for their mammalian counterparts [13,17,80,81].

Apoptosis is most often considered at the cellular level: cells that are unnecessary, damaged or diseased are removed by cell suicide. But it is essential to keep in mind that apoptosis promotes survival of the entire organism. It is certainly plausible that the same proteins that function as a rheostat for apoptosis also perform a similar function for survival, through energy modulation, in stressful life situations that are normally encountered by the organism.

## Methods

### Drosophila strains and cultures

All flies were raised on standard CY containing the standard 9% sucrose concentration (complete medium) at 25°C unless otherwise noted. For acute starvation conditions, larvae raised at low density in nutrient rich media were aged until early third instars then transferred to vials containing water soaked filter paper only. Protein-starvation media refers to 20% sucrose in agar. Larvae reared on limited nutrients were picked just after hatching and placed onto food containing 20% of the standard cornmeal/yeast media with normal agar concentration and either the standard concentration of sucrose (20% CY, 9% sucrose) or a reduced concentration of sucrose (20% CY, 1.8% sucrose). For rapamycin feeding, larvae were placed into petri dishes that contained the indicated concentrations of rapamycin mixed with standard food and food coloring. Food coloring was used to ensure larval uptake and mechanical mixing of rapamycin with food was performed to avoid chemical degradation. Somatic overexpression clones were generated in larvae with a 30 to 60 minute 37°C heat shock, 10 to 20 h prior to starvation and dissection.

Stocks used were: *daughterless-Gal4 *(*da-Gal4*) (Bloomington Stock Center, Indiana University, Bloomington, IN, USA), *UAS-TOR^TED ^*([82]; Bloomington Stock Center), *UAS-S6K^STDE ^*[50]; BSC), *buffy^H37 ^*[11]; BSC), *Tor*^Δ*P*^*P{neoFRT}*[36]*40A/CyO *([60]; BSC), *Cg*-*Gal4*, *UAS-GFP-LC3 *([43]; gift of Kim Finley, San Diego State University, San Diego, CA, USA), *Act > CD2 > Gal4*, *UAS-GFP III *(gift of T. Neufeld), *S6K^l-1 ^*([51]; gift of T Neufeld), *Atg1*^Δ*3D *^([22]; gift of T Neufeld), *UAS-mCherry-Atg8a *[83]; gift of Thomas Neufeld, University of Minnesota, Minneapolis, MN, USA), and *buffy^H37 ^*[11].

The *Atg1*^Δ*3D*^;*buffy^H37 ^*double mutant had a synthetic lethal phenotype: homozygous double mutant embryos hatched but died within 24 h of hatching. The very few double mutant larvae that survived past 48 h (5 larvae survived of 800 hatched larvae) were significantly smaller in size in comparison to their heterozygous siblings.

### Nile Red stain

Fat bodies from indicated larvae were dissected and fixed with 4% paraformaldehyde for 1 h. Fat bodies were washed three times for five minutes in phosphate-buffered saline (PBS) and incubated in 5 μM Nile Red in PBS for 1 h (with gentle agitation). After staining, fat bodies were washed and mounted in Vectashield (Vector Laboratories, Burlingame, CA, USA). For measurements of mean luminosity, care was taken to stain and observe dissected fat bodies identically. Measurements were made on images taken with identical exposure and camera settings and in which all staining was in the linear range. Note that these settings were necessarily different for Nile Red-stained fat bodies from 100% food-fed animals relative to animals fed on 20% food owing to the overall brightness of the stain in the fed animals. In order to keep the stains from fed animals in the linear range, a neutral density filter was used. Therefore, the luminosity data cannot be compared between 100% food and 20% food. Two identically-sized regions, encompassing only stained tissue, were chosen at random and the mean luminosity determined using the histogram function of Photoshop. The two regions were averaged and average data is presented for three individual animals. Lipid storage droplets (stained with Nile Red) were manually counted in 10 to 12 fat body cells for three individual animals and lipid droplet size in 33 to 43 fat body cells was determined using Photoshop. Nile Red staining of lysates was performed on pools of five third instar larvae lysed in radioimmunoprecipitation assay (RIPA) buffer and stained with Nile Red. Several dilutions were read at optical density (OD)_600 nm_.

For oenocyte staining, third instar larvae were pinned down onto a Sylgard (Corning Inc. Corning, NY, USA) dish, at their anterior and posterior ends in a droplet of PBS. Using a surgical razor and insect pins, the larvae were filleted such that the inner side of the integument was exposed. Filleted larvae with all internal organs removed were fixed in a droplet of 4% paraformaldehyde in PBS for 1 h. Fillets were then washed in PBS followed by a 30 minute incubation with 5 μM Nile Red in PBS.

### Caspase activity assay

Larvae were homogenized in a chilled glass mortar and pestle in a buffer containing 20 mM 4-(2-hydroxyethyl)-1-piperazine-ethanesulfonic acid (HEPES)-KOH (pH 7.5), 10 mM KCl, 1.5 mM MgCl_2_, 1 mM sodium ethylenediaminetetra-acetic acid (EDTA), 1 mM sodium ethylene glycol tetra-acetic acid (EGTA), 1 mM dithiothreitol, 0.1 mM phenylmethylsulfonyl fluoride (PMSF), and 0.5% Triton-X 100. Homogenates were spun at max speed for 10 minutes. A total of 50 μg of protein extract from the aqueous fraction was incubated with 50 μM Z-DEVD-AMC (Calbiochem EMD Millipore) substrate in a final volume of 100 μl in a 96-well plate. Fluorescence was monitored over time with excitation of 375 nm and emission of 450 nm using a SpectraMax GemimiXS (Molecular Devices, Sunnyvale, CA, USA) plate reader.

### Lysotracker staining

Larvae were washed in wells containing H_2_O, 96% ethanol and H_2_O, in that order. Clean larvae were then dissected and inverted, exposing fat body, in PBS and then incubated in 100 μM LysoTracker Red DND-99 or LysoTracker Green DND-25 (Molecular Probes/Life Technologies, Grand Island, NY, USA) as a whole carcass for 10 to 20 s. Inverted carcasses were then washed in PBS and placed onto a droplet of Vectashield (Vector Laboratories) containing 4',6-diamidino-2-phenylindole (DAPI), for fat body separation and mounting. Samples were imaged immediately.

### Fluorescence and transmission electron microscopy

All fluorescence microscopy (except mCherry-Atg8a) was performed on a Zeiss Axiovert 200 widefield microscope equipped with a mercury bulb, relevant filters and a Hamamatsu ORCA-ER CCD camera. Images were cropped and levels adjusted using Photoshop. Genotypes containing the *UAS-mCherry-Atg8a *transgene were treated as described above except imaged on a Leica SP5 confocal microscope using a 20 × 0.7 objective and 561 (mCherry) and 496 (LysoTracker Green DND 26, Life Technologies) laser lines for excitation. Emission bandwidths from beginning to end were: 493 nm to 520 nm (LTR-Green) and 566 nm to 700 nm (mCherry). Final images scanned using a line average of four.

For TEM, larvae were dissected and inverted to expose fat body and fixed overnight in 2% paraformaldehyde, 4% glutaraldehyde, 2% sucrose and PBS at 4°C. Fixed tissue was then rinsed in PBS and post fixed in 2% osmium tetroxide and PBS overnight at 4°C. Carcasses were dehydrated with ethanol and dissected to obtain fat body. Fat body was then embedded in epoxy resin, incubated in propylene oxide 2 × for 20 minutes, followed by a 50:50 propylene oxide and epoxy resin incubation overnight in a vacuum. The following day samples were treated with 100% epoxy resin for 2 h in a vacuum, and transferred to a capsule filled with resin followed by a 24 to 48 h incubation at 60°C. Tissue blocks were then trimmed using a single-edged razor blade under a dissecting microscope (Nikon Instruments). A short series of ultrathin (60 to 80 nm) sections containing fat body were cut from each block with an ultramicrotome (Reichert-Jung) and sequential sections were collected on mesh and formvar-coated slot grids. The sections were stained with uranylacetate and lead citrate to enhance contrast. Sections were examined with a Philips CM-10 transmission electron microscope and images were captured with a Gatan digital camera.

### Quantification of autophagic structures, cell size and pupal mass

For TEM images, autolysosomes were quantified from over 60 images, each with a field view of about 40 μm^2 ^at a magnification of 2950 ×. A total of five or six larvae are represented per genotype or nutrient condition. For lysotracker staining, punctae were counted using MetaMorph from at least five representative images from five to seven different animals. For measurement of fat body cell size, fat bodies from random positions within the larva were stained with Nile Red and imaged. Individual fat body cells were outlined manually and the perimeter and area were measured using Photoshop. The perimeter is reported. To determine pupal mass, clean tan pupae (within a 24 h age window) were picked from the side of vials and weighed in groups of 5.

### Immunoblot analysis

Pooled larvae were homogenized in a chilled buffer containing 1% NP-40, 150 mM sodium chloride, 10 mM sodium phosphate pH 7.2, 50 mM sodium fluoride, with freshly added 100 μM PMSF, 100 nM okadaic acid, 0.2 mM sodium vanadate and protease inhibitors. An aliquot was removed for bicinchoninic acid (BCA) analysis (Pierce, Thermo Scientific, Rockford, IL, USA), while the remaining homogenate was treated with gel loading buffer, heated at 90°C for 5 minutes, spun and loaded. For the detection of S6K, a 10% sodium dodecyl sulfate polyacrylamide gel electrophoresis (SDS-PAGE) gel was used, transferred onto a polyvinylidene fluoride (PVDF) membrane (EMD Millipore) and probed with either rabbit S6K-phospho-T398 S6K (Cell Signaling Technology, Danvers, MA, USA), rabbit S6K-phospho-T229 (mammalian) (Cell Signaling Technology) or rabbit anti-S6K (gift from T Neufeld) all at 1:1,000. β-Tubulin (E7) antibody (Developmental Studies Hybridoma Bank, University of Iowa, Iowa City, IA, USA) was used at 1:4,000 and served as our loading control. For the detection of 4E-BP, a 15% SDS-PAGE gel was used and transferred onto a nitrocellulose membrane (Whatman, GE) with a 0.2 μm pore size. Antibody 1868 to 4E-BP was a gift from Nahum Sonenberg (McGill University, Montreal, Quebec, Canada) [84] and used at 1:1,000. Rabbit secondary antibodies conjugated to horseradish peroxidase (Vector Laboratories) were used at 1:10,000. For quantification, film was scanned and bands quantified using ImageJ. For quantification of anti-tubulin loading control, mouse AlexaFluor-800 nm secondary conjugates were used at 1:10,000 and an Odyssey InfraRed Imaging System and software (Li-Cor BioSciences, Lincoln, NB, USA) were used to analyze the intensity of bands according to manufacturer's instructions.

### Biochemical assays

Total triacylglycerides were measured from 20 staged larvae. Pooled larvae were homogenized in PBT (0.05% Tween-20 in PBS) using a Kontes pestle (30 strokes). An aliquot was taken for protein determination (BCA assay, Thermo) while the rest was incubated at 70°C for 5 minutes. Heat-treated samples were spun at max speed in a table-top centrifuge. A fraction of the homogenate was used for the Thermo Infinity Triglyceride Kit (Thermo) following the manufacturer's protocol. Readings were normalized to protein content. For lactate determination, 30 staged larvae were homogenized in 100 μl of PBS (no detergent) using a Kontes pestle (30 strokes). A sample aliquot was taken for protein determination and the rest subjected to heat treatment (60°C for 15 minutes) and spun. A total of 10 μl of homogenate was used for Lactate Assay Kit (Biovision cat no. K607-100, Milpitas, CA, USA) following the manufacturer's protocol. Readings were normalized to protein content. For glycogen determination, 10 larvae were homogenized on ice with 200 μl of chilled ultra-pure water. An aliquot was quickly removed and flash frozen while the rest of the lysates was immediately placed in a boiling water bath for 5 minutes (all steps occurring within a minute from first stroke of homogenization). Samples were then spun for 10 minutes at high speed and supernatant was used for Glycogen Assay Kit (Biovision cat no. K646-100) at a tenfold dilution following the manufacturer's protocol. For ATP analysis, pooled larvae (10 larvae/200 μl buffer) were homogenized in 1 × Reporter lysis buffer (Promega, Madison, WI, USA), and immediately flash frozen. The frozen samples were boiled for 15 minutes to destroy ATPase activity, then spun at 17 800 *g *for 5 minutes and the supernatant was diluted 100-fold with the same buffer. The cellular ATP content was quantified by a luciferin/luciferase-based assay using an ATP Determination Kit (Sigma-Aldrich). Luminofluorescence was measured using the Wallac ARVO SX 1420 Multilabel Counter (Perkin Elmer Life Sciences, Waltham, MA, USA) and the data were normalized to the protein content.

## Competing interests

The authors declare that they have no competing interests.

## Authors' contributions

JPM and CBB designed and interpreted all the results of the experiments. JPM carried out all experiments except for the following: MY-YC carried out the triacylglyceride assays and participated in the immunoblotting and feeding experiments, CBB carried out oenocyte staining, some genetic crosses and quantification of results. The manuscript was initially drafted by JPM and written by CBB. All authors read and approved the final manuscript.

## Supplementary Material

Additional file 1 **Figure S1**. **(A) **Semiquantitative reverse transcription polymerase chain reaction (RT-PCR) demonstrating knockdown of *buffy *transcript levels in two lines in which *buffy *RNAi was expressed using the ubiquitous driver *daughterless*-*Gal4*. (**B-D) **Fat body Nile Red stain of RNAi lines reared in restrictive media (20% cornmeal/yeast/agar food (CY); 1.8% sucrose, compare to Figure 1D) phenocopies observations made in the *buffy^H37 ^*null mutant. (B) *da-Gal4 *driver alone; (C) *da*-*Gal4*, *UAS-buffyRNAi498*; (D) *da*-*Gal4*, *UAS-buffyRNAi499*. **(E-G) **LysoTracker Red (LTR) stain on fat bodies from RNAi lines amino-acid starved for 2 h also phenocopies the *buffy^H37 ^*null mutant. (E) *da*-*Gal4 *driver alone; (F) *da*-*Gal4*, *UAS-buffyRNAi498*; (G) *da*-*Gal4*, *UAS-buffyRNAi499 *(compare to Figure 4A). Click here for file

Additional file 2 **Figure S2**. Immunoblot using phosphospecific S6K antibody conducted on larval lysates from *buffy *knockdown lines. RNAi line 499 reproduced the increase in phosphorylated S6K that is observed in the *buffy *null allele. Click here for file

Additional file 3 **Figure S3**. **(A) **Quantification of LysoTracker Red (LTR) stain from three different wild-type alleles to determine variation in autophagic responses to 2 h of starvation. Graph represents numbers from 11 different animals. The *buffy *mutant is included for comparison. **(B) **LC3-green fluorescent protein (GFP) marker highlights autophagic vacuoles to corroborate LTR data. Shown are fat bodies from 2 h-starved larvae expressing LC3-GFP (green, middle panels) and stained with LTR (red, top panels) with merged images shown in the bottom panels. More LC3-GFP punctae are observed in the *buffy^H37 ^*mutant and wild-type larvae starved for 2 h. Yellow arrowheads point to examples of LC3-GFP punctae that colocalize with LTR (insets in wild-type images), indicating autolysosomes. Genotypes for (B): *cg-Gal4*, *UAS-LC3-GFP *and *cg-Gal4*, *UAS-LC3-GFP*, *buffy^H37^*. Click here for file

Additional file 4 **Figure S4**. LysoTracker Red (LTR) stain (red in left panels, grayscale in right panels) of single cell clones overexpressing dominant negative *Tor *(*Tor^TED^*, green) in the *buffy^H37 ^*mutant and wild-type background. Clones of *Tor^TED ^*in wild-type fat body **(A) **or *buffy^H37 ^*fat body **(B) **from fed larvae are autophagic and small as expected. Similarly, clones of *Tor^TED ^*in wild-type fat body **(C) **or *buffy^H37 ^*fat body **(D) **from larvae starved for 2 h are indistinguishable in their autophagic response. Note that in both cases, *Tor^TED ^*cells have the same amount of autophagy as neighboring *Tor+ *cells in the starved conditions. The *Tor^TED ^*clones observed in (C) and (D) were generated later in development as opposed to those observed in (A) and (B), which accounts for the difference is cell size. Images are representative of 7 to 10 different animals surveyed per genotype. Genotypes: **(D, F) ***hs flp*/+*; UAS-Tor^TED^/+; Act > CD2 > Gal4*, *UAS-GFP/+*. **(E, G) ***hs flp/+; buffy^H37 ^, UAS-Tor^TED^/buffy^H37^; Act > CD2 > Gal4*, *UAS-GFP/+*. Click here for file
