# Supplementary material for: Drosophila larvae lacking the bcl-2 gene, buffy, are sensitive to nutrient stress, maintain increased basal target of rapamycin (Tor) signaling and exhibit characteristics of altered basal energy metabolism
Source: BMC Biol. 2012 Jul 24;10:63. doi: 10.1186/1741-7007-10-63 (PMC3411425; doi:10.1186/1741-7007-10-63)
Supplement: Additional file 1 — Figure S1. (A) Semiquantitative reverse transcription polymerase chain reaction (RT-PCR) demonstrating knockdown of buffy transcript levels in two lines in which buffy RNAi was expressed using the ubiquitous driver daughterless-Gal4. (B-D) Fat body Nile Red stain of RNAi lines reared in restrictive media (20% cornmeal/yeast/agar food (CY); 1.8% sucrose, compare to Figure 1D) phenocopies observations made in the buffyH37 null mutant. (B) da-Gal4 driver alone; (C) da-Gal4, UAS-buffyRNAi498; (D) da-Gal4, UAS-buffyRNAi499. (E-G) LysoTracker Red (LTR) stain on fat bodies from RNAi lines amino-acid starved for 2 h also phenocopies the buffyH37 null mutant. (E) da-Gal4 driver alone; (F) da-Gal4, UAS-buffyRNAi498; (G) da-Gal4, UAS-buffyRNAi499 (compare to Figure 4A). [file 1741-7007-10-63-S1.PDF]

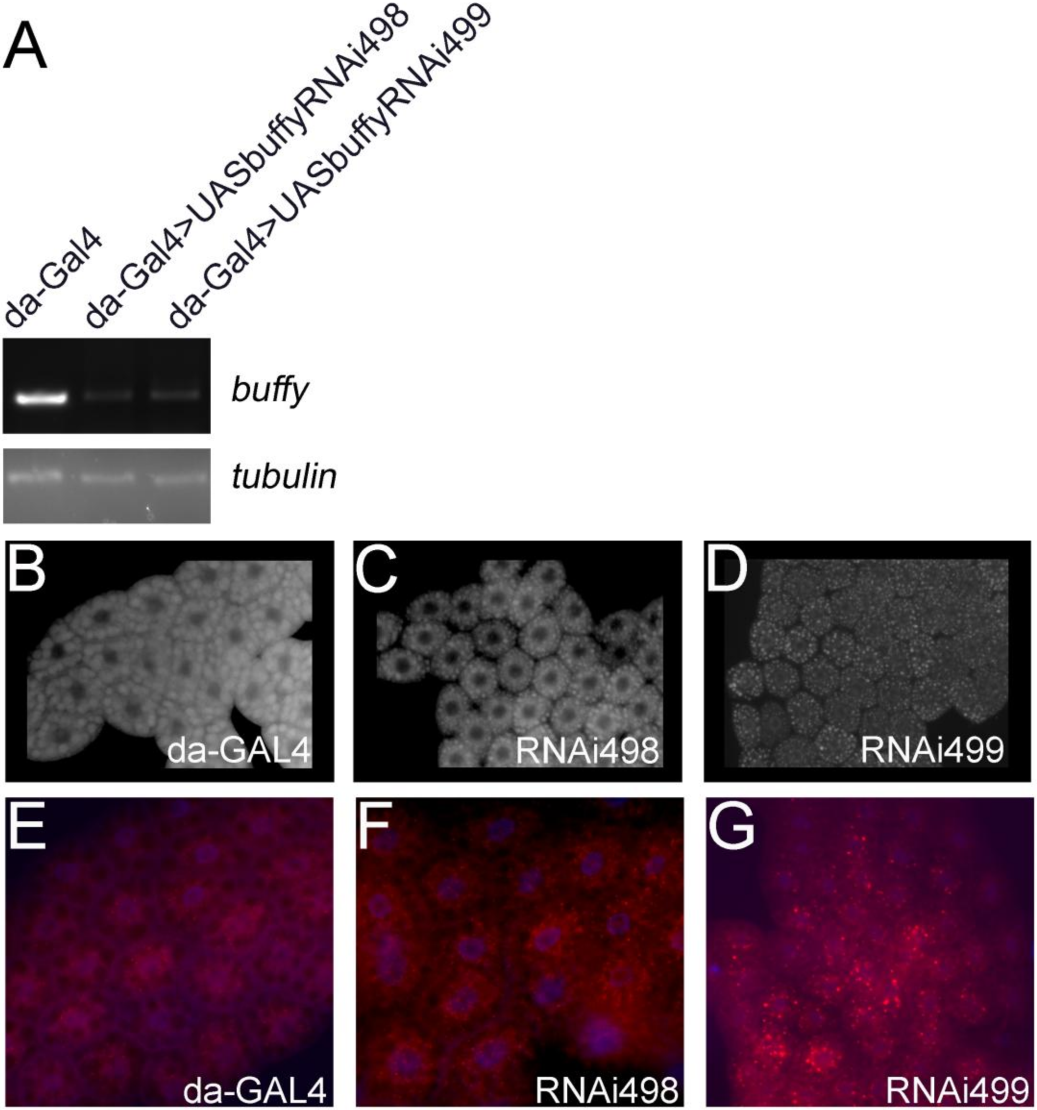
A
da-Gal4
da-Gal4>UASbuffyRNAi498
da-Gal4>UASbuffyRNAi499
buffy
tubulin
B
da-GAL4
C
RNAi498
D
RNAi499
E
da-GAL4
F
RNAi498
G
RNAi499
