# Supplementary figures and images for: Drosophila larvae lacking the bcl-2 gene, buffy, are sensitive to nutrient stress, maintain increased basal target of rapamycin (Tor) signaling and exhibit characteristics of altered basal energy metabolism
Source: BMC Biol. 2012 Jul 24;10:63. doi: 10.1186/1741-7007-10-63 (PMC3411425; doi:10.1186/1741-7007-10-63)

da-GAL4

da-GAL4>UASbuffyRNAi 496

da-GAL4>UASbuffyRNAi 498

da-GAL4>UASbuffyRNAi 499

75

50

anti-S6K<sup>pT398</sup>

Tubulin

1.0

0.5

1.3

2.5

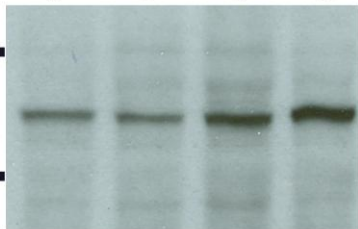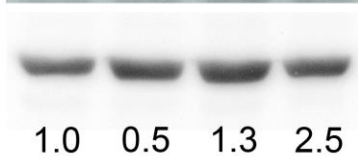

Supplement: Additional file 2 — Figure S2. Immunoblot using phosphospecific S6K antibody conducted on larval lysates from buffy knockdown lines. RNAi line 499 reproduced the increase in phosphorylated S6K that is observed in the buffy null allele. [file 1741-7007-10-63-S2.PDF]

**A**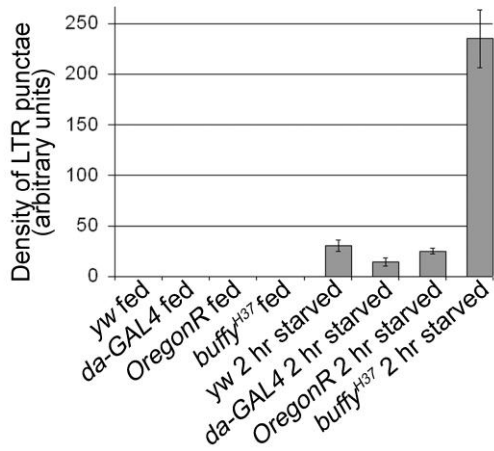**B**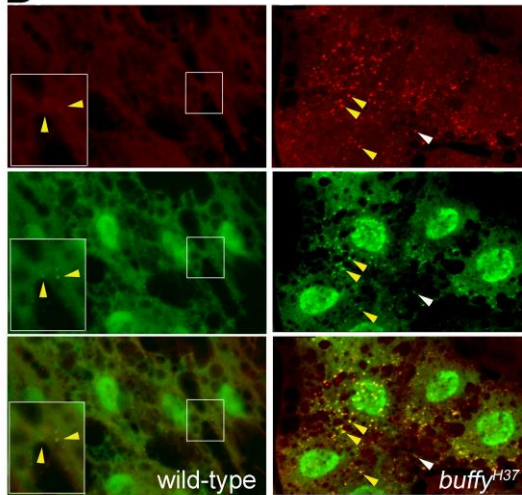

Supplement: Additional file 3 — Figure S3. (A) Quantification of LysoTracker Red (LTR) stain from three different wild-type alleles to determine variation in autophagic responses to 2 h of starvation. Graph represents numbers from 11 different animals. The buffy mutant is included for comparison. (B) LC3-green fluorescent protein (GFP) marker highlights autophagic vacuoles to corroborate LTR data. Shown are fat bodies from 2 h-starved larvae expressing LC3-GFP (green, middle panels) and stained with LTR (red, top panels) with merged images shown in the bottom panels. More LC3-GFP punctae are observed in the buffyH37 mutant and wild-type larvae starved for 2 h. Yellow arrowheads point to examples of LC3-GFP punctae that colocalize with LTR (insets in wild-type images), indicating autolysosomes. Genotypes for (B): cg-Gal4, UAS-LC3-GFP and cg-Gal4, UAS-LC3-GFP, buffyH37. [file 1741-7007-10-63-S3.PDF]

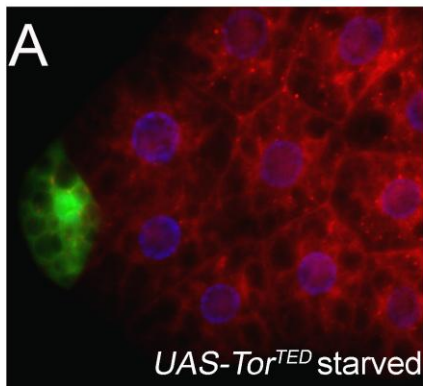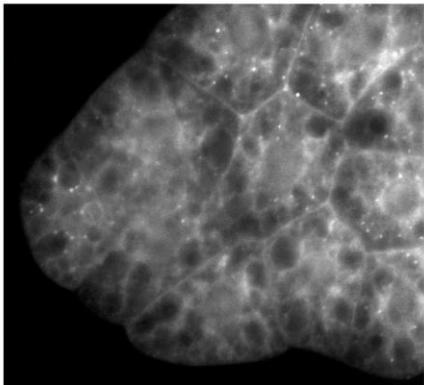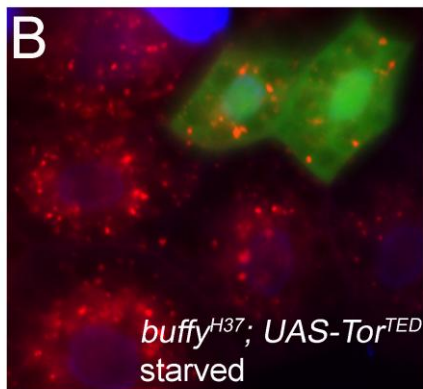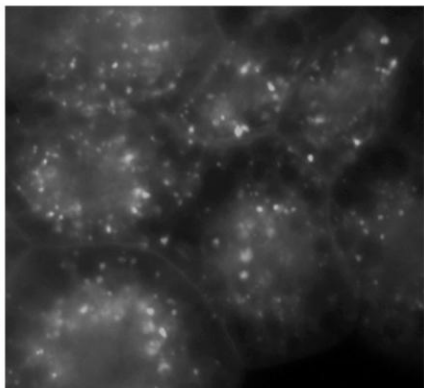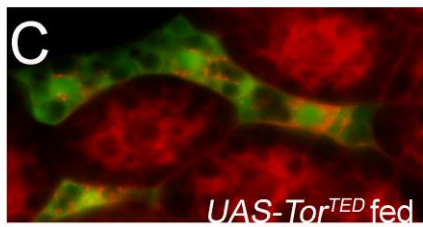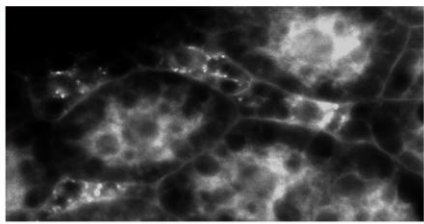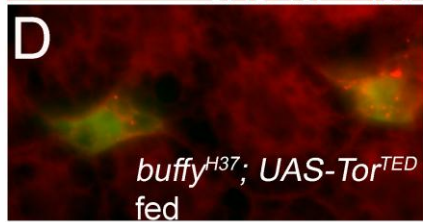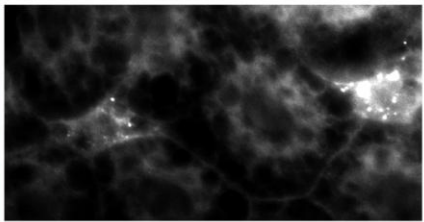

Supplement: Additional file 4 — Figure S4. LysoTracker Red (LTR) stain (red in left panels, grayscale in right panels) of single cell clones overexpressing dominant negative Tor (TorTED, green) in the buffyH37 mutant and wild-type background. Clones of TorTED in wild-type fat body (A) or buffyH37 fat body (B) from fed larvae are autophagic and small as expected. Similarly, clones of TorTED in wild-type fat body (C) or buffyH37 fat body (D) from larvae starved for 2 h are indistinguishable in their autophagic response. Note that in both cases, TorTED cells have the same amount of autophagy as neighboring Tor+ cells in the starved conditions. The TorTED clones observed in (C) and (D) were generated later in development as opposed to those observed in (A) and (B), which accounts for the difference is cell size. Images are representative of 7 to 10 different animals surveyed per genotype. Genotypes: (D, F) hs flp/+; UAS-TorTED/+; Act > CD2 > Gal4, UAS-GFP/+. (E, G) hs flp/+; buffyH37 , UAS-TorTED/buffyH37; Act > CD2 > Gal4, UAS-GFP/+. [file 1741-7007-10-63-S4.PDF]
